# Parametrisation of a DEM model for railway ballast under different load cases

**DOI:** 10.1007/s10035-017-0740-7

**Published:** 2017-08-02

**Authors:** Bettina Suhr, Klaus Six

**Affiliations:** Virtual Vehicle Research Center, Inffeldgasse 21/A, A-8010 Graz, Austria

**Keywords:** DEM simulation, Contact modelling, Parametrisation, Railway ballast, Tribology

## Abstract

The prediction quality of discrete element method (DEM) models for railway ballast can be expected to depend on three points: the geometry representation of the single particles, the used contact models and the parametrisation using principal experiments. This works aims at a balanced approach, where none of the points is addressed with excessive depth. In a first step, a simple geometry representation is chosen and the simplified Hertz–Mindlin contact model is used. When experimental data of cyclic compression tests and monotonic direct shear tests are considered, the model can be parametrised to fit either one of the two tests, but not both with the same set of parameters. Similar problems can be found in literature for monotonic and cyclic triaxial tests of railway ballast. In this work, the comparison between experiment and simulation is conducted using the entire data of the test, e.g. shear force over shear path curve from the direct shear test. In addition to a visual comparison of the results also quantitative errors based on the sum of squares are defined. To improve the fit of the DEM model to both types of experiments, an extension on the Hertz–Mindlin contact law is used, which introduces additional physical effects (e.g. breakage of edges or yielding). This model introduces two extra material parameters and is successfully parametrised. Using only one set of parameters, the results of the DEM simulation are in good accordance with both experimental cyclic compression test and monotonic directs shear test.

## Introduction

This work focuses on the behaviour of railway ballast under general monotonic and cyclic loading conditions. To describe and predict the complex behaviour of such solid-like granular materials the discrete element method (DEM) is a very popular particle-based method. Before any simulation, several choices for modelling the considered granular material have to be made. At first, a representation of the geometry of the single particles is necessary. Second, models for contact forces in normal and tangential direction have to be chosen introducing several material parameters. Finally, the parametrisation of the contact models and the validation of the whole DEM model have to be done, which are challenging tasks. Regarding these aspects a well-balanced approach should be chosen. For example, a very detailed representation of the particle geometry will probably not improve the prediction quality of the DEM model while still applying very simple contact laws neglecting relevant physical phenomena and vice versa.

For railway ballast, DEM model validation is often carried out using principal experiments like compression tests, direct shear tests, triaxial tests or box tests, compare e.g. [1–7]. The more principal experiments are used for DEM model parametrisation and validation the higher the prediction quality will be when applying these models to real railway situations (e.g. cyclic compression and shearing behaviour of ballast under sleepers in curved tracks). State of the art DEM modelling often has problems to describe different principle experiments with one set of model parameters. This can result in a poor prediction quality when applying these models to realistic and thus more complex situations.

In this work, a well-balanced approach between geometry representation and contact modelling is followed. For computational efficiency, the particle’s geometry is represented in a very simple way. Contact modelling starts with the simplified Hertz–Mindlin law and is then extended to include additional physical phenomena (e.g. breakage of edges or yielding), to bring the simulation into accordance with the chosen principal experiments. Furthermore, a methodology is presented taking into account the whole data of principal experiments, e.g. shear-force over shear-path from a direct shear box experiment, instead of using single data characteristics only, e.g. bulk friction angle. This is very important for modelling railway ballast. In the compression test, the information of settlement between cycles and the shape of the single loading loops is lost, when only the bulk stiffness is considered. In the direct shear test, the initial stiffness of the shear force–shear path curve is important for track stiffness. This information is not included in the bulk friction angle. Therefore, in this work the entire curves are compared visually and quantitative error measures are introduced, which are based on the sum of squares. This methodology is applied to data from cyclic compression tests and direct shear tests taken from the literature showing its capability.

This work is organised as follows. Section 2 starts with a literature overview. Section 3 summarises experimental results of compression and direct shear tests, taken from literature [5], which will be used for model parametrisation. DEM simulations of these experiments using the simplified Hertz–Mindlin contact model are presented in the next section. To be able to simulate both cyclic compression test and monotonic direct shear test with only one set of parameters, the Hertz–Mindlin model is extended to the so called Conical damage model presented in [6]. In Sect. 5 this contact model is given in a slight modification and an efficient algorithm for its accurate solution will be presented. In a parameter study in the following Section, the influence of the model parameters on the bulk behaviour in both compression and direct shear test is investigated. Section 7 deals with the comparison of simulation results obtained with the parametrised model and the experimental results. In this case, both the simulations of the compression tests and the direct shear tests are conducted with only one set of parameters and good agreement between simulations and experiments is obtained. Conclusions are drawn in the last Section.

## Literature overview

The behaviour of coarse and angular granular material, such as railway ballast or crushed rock, is a topic of intensive research both on experimental and on simulation side.

Regarding particle shape modelling, several different approaches are used for railway ballast or crushed rock. Rigid clumps of spheres allow an approximation of complex shaped particles, while the same contact laws as for single spheres can be used. This approach is used, e.g.  by [2, 4, 5, 7, 8]. In [4], Laryea et al. compared results of simulation with complex particle shapes to those using a simple two ball clump. For the considered box tests, the simplified clumps were able to yield qualitatively similar results. In [5], Coetzee investigated the modelling of crushed rock with simple clumps (two spheres) or more complex clumps (up to eight spheres). The results of compression test or direct shear test simulations, which Coetzee obtained with the simple clumps were comparable to those obtained with complex clumps. When particle breakage is considered, also clumps of bonded spheres are used, see e.g. [9, 10]. Apart form clumps, also polyhedra are used to model railway ballast, see e.g. [1, 11, 12]. In the approach used by Tutumluer et al. [11] and Quian et al. [1], data from 3d-scanned ballast stones can be used to build polyhedral DEM particles. In [13] Ahmed et al. and in [6] Harkness et al., so called potential particles for the simulation of triaxial tests of railway ballast are used. Ahmed et al. present a method to manually adapt the shape of a potential particle to the shape of a ballast stone.

Which contact model is used in DEM simulations of course depends on the chosen geometry representation. For polyhedral particles, the contact forces are often related to the intersecting volume of the contact and linear springs are used for the calculation of normal and tangential contact forces. When clumps of spheres are used, commonly used models are the linear spring model of Cundall and Strack [14], or the simplified Hertz–Mindlin model, see e.g. [15]. In contact normal direction, the Hertz model provides the analytic solution for elastic contacts between two spheres or a sphere and a plane. In tangential direction, the simplified Mindlin model together with Coulomb’s law provides a trade-off between an accurate solution and computational efficiency. Despite these two advantages of the Hertz–Mindlin model, there are also some problems, which might occur in some simulations. It is reported in literature that in simulations of the direct shear test the bulk shear stiffness is too high (using the original material parameter of the particles), see [16] and references therein. In the same work [16], Härtl and Ooi conducted direct shear tests on paired glass spheres (two glued spheres). They reported a dependency of the bulk friction angle on the applied normal pressure. In their simulations, using the Hertz–Mindlin model, this effect could not be reproduced. The authors of this work succeeded in simulating the pressure dependency of the bulk friction angle in [17]. Motivated by tribological considerations, they introduced a pressure dependency in the individual interparticle friction coefficients, thus modifying Coulomb’s law.

In [6], Harkness et al. conducted simulations of monotone and cyclic triaxial tests on railway ballast using potential particles. They found that the Hertz–Mindlin model was not able to fulfil the “simultaneous requirements of an apparently low initial stiffness in monotonic loading at high confining pressures (through crushing of the asperity), while retaining high elastic stiffness for the case of cyclic loading, which is of crucial importance in modelling railway ballast” [6]. To overcome this problem, they introduced a modified contact model: the conical damage model (CDM). A detailed description will be given later in this work. In the literature, there are many examples where the simplified Hertz–Mindlin model is successfully used to model granular material. However, in the special situations mentioned above, problems occur and modifications are necessary.

Whatever contact model is chosen in a simulation, its parametrisation and the DEM model validation remains usually a challenge. Coetzee gives a good overview over used approaches in [5]. Often parametrisation and validation of DEM model are conducted via the comparison of simulation results with principal experiments, e.g. compression tests, direct shear tests or triaxial tests. Often only one type of principal experiment is used. However, a DEM model validated using different types of principal experiments can be considered more trustworthy in simulating other load cases. For the comparison between DEM simulations and measurements mainly two approaches exist in the literature. One group of authors compares the two curves visually (using the entire data). Another group computes certain characteristics from the curves and compares theses, e.g. bulk stiffness from a compression test. A comparison based on characteristics only can be problematic, as will be discussed later in this work.

## Principal experiments

In [5], Coetzee conducted cyclic compression tests and direct shear tests on crushed rock. As these results will be used in the current work, a short summary of the experiments will be given, while the reader is referred to the original work for details. Both compression and shear tests were conducted in a cylindrical shear cell with a diameter of 590 mm and a height of 330 mm.The maximal particle size was 40 mm, details about the gradation are not given. When filling the shear cell, the resulting samples had porosities between 0.45 and 0.47 with an average of 0.46.

### Compression tests

**Fig. 1:**
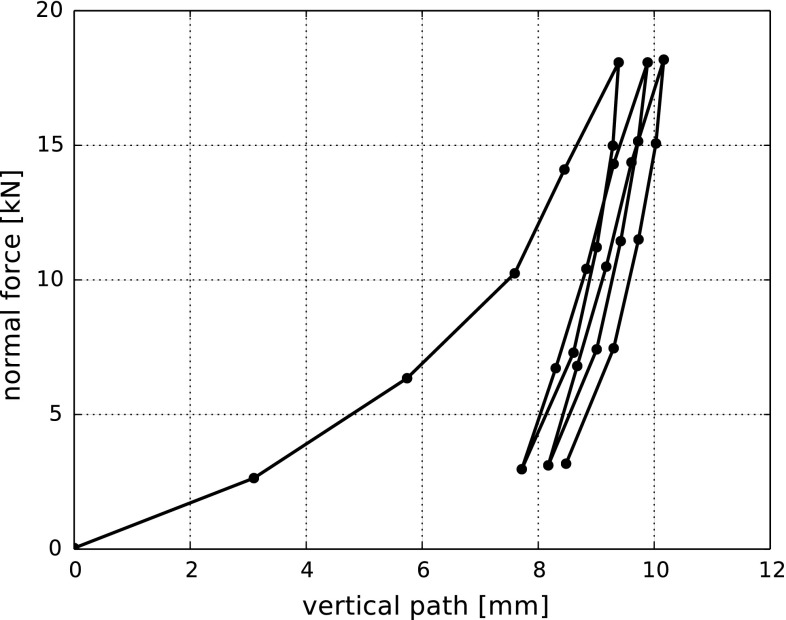
Compression test: path and normal force for the discrete levels of applied normal loads. Data taken from [5]

After filling the shear cell, a lid was placed on top of the sample. The normal load was applied via weights, which were increased stepwise until approximately 66 kPa were reached. Three loading-unloading cycles were conducted in each compression test. The bulk stiffness was calculated as average between the gradients of the second and third loading cycles of the stress-strain curve. Between which values of stress the gradient was fitted, was not specified. Coetzee obtained values of the bulk stiffness, *B*, ranged between 8.4 and 9.1 MPa with an average value of 8.7 MPa. Experimental results of a compression test are shown in Fig. 1 as normal force over path. This data is obtained from [5] by plot digitalisation and thus may contain small inaccuracies.

### Direct shear tests

**Fig. 2:**
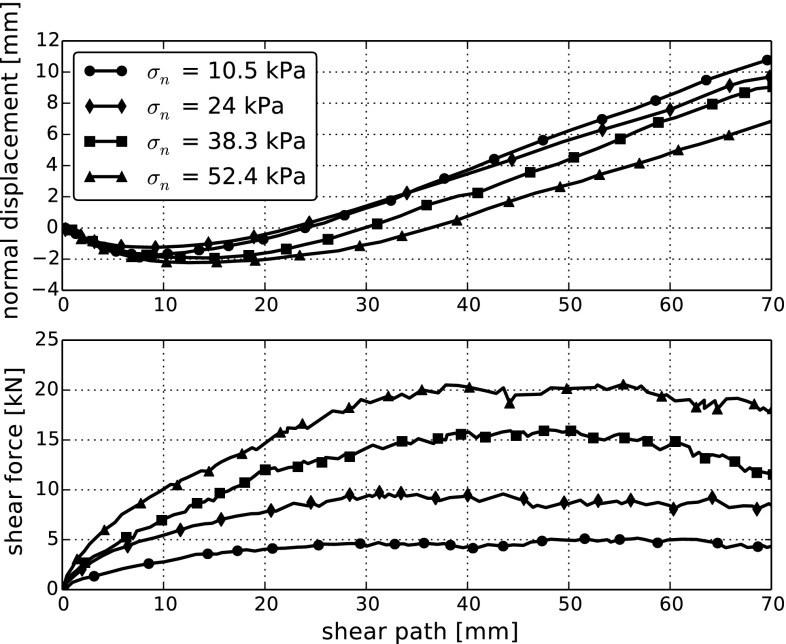
Shear force and dilation of direct shear tests, data taken from [5]

Direct shear tests were conducted with applied normal stresses of $\sigma _n = 10.5, 24, 38.8, 52.4$ kPa. The maximal shear path was 70 mm and shearing took place at a velocity of 1 mm/s. The bulk friction angle, $\phi$, calculated by Coetzee varied between $50.1^{\circ }$ and $56.3^{\circ }$ with an average of $52.8^{\circ }$. To calculate the angle of dilation $\psi$, a line was fitted through the dilation curve between 45 and 70 mm of displacement. Coetzee calculated values for $\psi$, which ranged between $11.2^{\circ }$ and $13.5^{\circ }$ with an average of $\psi =12.5^{\circ }$. Experimental results are shown in Fig. 2, which is obtained from [5] by plot digitalisation and thus may contain small inaccuracies.

**Fig. 3:**
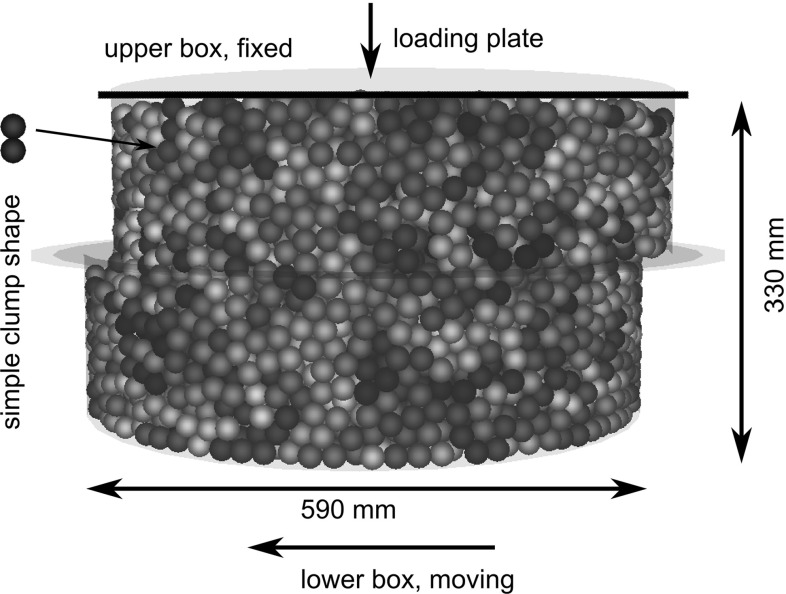
DEM model of direct shear test

## DEM simulations with Hertz–Mindlin contact model

The DEM simulations presented in this work are carried out with the Open-Source software Yade [18]. In this software the soft contact approach is used together with explicit discretisation in time. The shear cell is modelled in its original size. As already mentioned, in this work the crushed rock will be represented in a simple way. Rigid clumps of two equi-sized spheres with a constant aspect ratio of two (i.e. no overlap between the spheres) will be used. The clumps are all of the same size and the spheres in the clump have a radius of 10 mm. Thus, the maximal clump axis is 40 mm and has the same length as the maximal particle size in the experiments. The ratio between the diameter of the shear box and the longest particle axis is bigger than 10. In Fig. 3, a picture of a simulated shear test at a shear path of 30 mm is given.

Of course, the chosen clump shape is a strong simplification of the complex shaped crushed rock and much more detailed shape representations are used in the literature, compare Sect. 2. However, even in these (computationally demanding) DEM models with detailed shape representations some degree of simplification of the reality has to be accepted. For example, effects resulting from the macroscopic particle texture cannot be considered in the particles’ shape and are thus shifted into the contact modelling (e.g. adaption of coefficient of friction). The same holds true for the approach chosen in this work. Particle shape is kept very simple but the clumps can provide some interlocking between particles (in contrast to single spheres). The simplifications of the real particle shape and errors resulting from this simplification can be compensated, to some extent, by adapting the particle-particle contact law parameters. Obviously this approach can only be successful, if the geometry is not overly simplified and the considered contact law takes into account all relevant physical effects.

**Table 1:** Material parameters for DEM simulations using the Hertz–Mindlin model

Particles: rock	
Sphere diameter	10 mm
Density	$2600~\hbox {kg/m}^3$
Poissons ratio	0.2
Young’s modulus	50–600 MPa
Friction coefficient (rock–rock)	0.3–0.5
Walls: steel	
Density	$7834~\hbox {kg/m}^3$
Poissons ratio	0.28
Young’s modulus	200 GPa
Friction coefficient (rock–steel)	0.2

Simplified geometries were successfully used to model railway ballast in literature. In [4], Laryea et al. used clumps consisting of two overlapping spheres of different radii and obtained qualitatively similar results compared to simulations using a more complex particle shape representation. In [5], Coetzee simulated railway ballast in compression tests and direct shear tests and compared samples which consisted of clumps of two, four and eight spheres, respectively. For each clump type, the parameters of the contact law were calibrated individually. Using these different parameters for each clump type, a good fit to the experimental data was achieved for all clump types, thus also for the simplest clumps of two spheres. The comparison between experiments and simulations is based on bulk stiffness in the compression test and bulk friction angle and angle of dilatancy in the direct shear test, force-path curves are not shown. Nevertheless, Coetzee’s work [5], shows that some differences in particle shape can be compensated by the adaption of contact law parameters. This holds true as long as no oversimplification of the particle shape is made, e.g. usage of simple spheres, which is also shown in [5] for comparison. The investigation of the influence of simplified particle shape on the found contact law parameters and their relation to measured material parameters, is an interesting topic and remains for future work.

In this section, the simplified Hertz–Mindlin no-slip contact model, compare e.g. [15], will be used and no damping is applied. In the Hertz–Mindlin model, the most important material parameters are the Young’s modulus and the friction coefficient of the modelled rock material. They will be varied for the parametrisation of the DEM model. All material parameters used are given in Table 1.

For sample generation, clumps were created in a box above the shear cell and were allowed to settle due to gravity. All particles which were not entirely inside the shear cell were deleted. To control the porosity of the sample the friction coefficient was reduced in this phase of the simulation. In this work, four different initial configurations are used. The samples consisted of 5850 clumps on average and the porosity was between 0.456 and 0.458. The experimental samples had porosities between 0.45 and 0.47 with an average of 0.46. Thus, the porosity of the simulated samples scattered less than in the experiments and the values were in the lower range of the experimental samples.

**Fig. 4:**
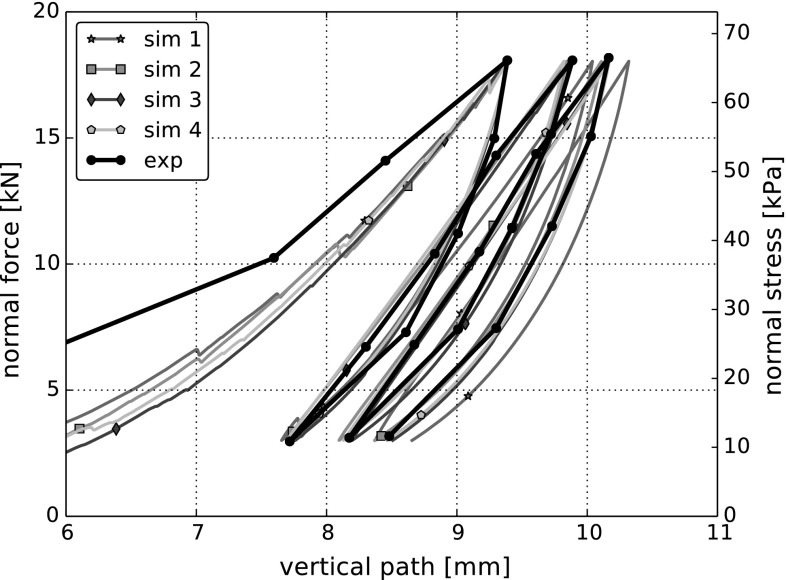
DEM simulations of compression tests using the Hertz–Mindlin model for four different initial configurations and experimental data, taken from [5]. For all simulations parameter values $E=600~\hbox {MPa}$ and $\mu =0.3$ were used. Note that simulation results are shifted horizontally, for better comparison with the experiments

**Table 2:** Simulations of the compression tests using the Hertz–Mindlin model with different initial configurations: relative sum of squares error, $\varepsilon _p$, and bulk stiffness, B. For comparison, the bulk stiffness calculated from the experimental data is also given

Initial conf.	1	2	3	4	Exp.
$\varepsilon _p$ [−]	$2.2\cdot 10^{-2}$	$4.1\cdot 10^{-3}$	$6.3\cdot 10^{-3}$	$5.4\cdot 10^{-3}$	−
B [MPa]	8.6	8.7	8.8	8.7	9.4

### Compression tests

For the simulation of the compression test a steel plate was inserted above the spheres. This loading plate moved downwards with a constant velocity of 0.5 mm/s until the maximal stress of 66 kPa was reached. The plate then moved upwards with the same speed until the minimal stress of 11 kPa was reached. In this way, three loading-unloading cycles were simulated, analogue to the experimental data.

The choice of the model parameters for rock *E* and $\mu$ was not difficult in this case. An increase of *E* caused an increase in the obtained bulk stiffness. While the influence of $\mu$ on the bulk stiffness was small, an increase of $\mu$ changed the shape of the single loading-unloading loops and reduced the enclosed area. Due to the strong simplifications in particle geometry representation, it is not likely that the obtained parameter values will be close to literature values for rock or ballast. In Fig. 4 simulation results for four different initial configurations are shown. The chosen parameter values are $E=600~\hbox {MPa}$ and $\mu =0.3$. A very good fit of simulations to the experimental data are obtained regarding the bulk stiffness, the increment in vertical path between the load cycles (settlement) and the shape of the single loading-unloading loops. The scattering in results caused by the different initial configurations is small, with exception of the first initial setting. Here, the bulk stiffness is slightly lower than in the other simulations. It is important to note that the simulation results in Fig. 4 are shifted horizontally, such that the vertical path belonging to the first force maximum, corresponds to the experimental one. The initial vertical path can be expected to show a strong scattering in experiments, even when the same procedure is used for assembling the samples. Therefore, this initial path is ignored in the following plots and in the error computation.

To obtain a quantitative comparison between simulation and experimental results, a relative error based on the sum of squares will be defined. As the compression test is a force-controlled experiment, this error will be calculated with respect to the vertical path. The data is split at the normal force minima and maxima. The obtained separate loading or unloading parts of the data have a unique mapping of the force *f* to the vertical path *p*(*f*). Denoting the path in the experiment and simulation with $p^{exp}(f)$ and $p^{sim}(f)$ respectively, for the second loading in the test the difference between simulation and experiment, $\varepsilon _{diff}^{l,2}$, and the experimental values, $\varepsilon _{exp}^{l,2}$, is calculated as follows:

1a $$\begin{aligned} \varepsilon _{diff}^{l,2}&= \sum _{f=f_{min}}^{f_{max}} \left( p^{exp}(f) - p^{sim}(f)\right) ^2 \end{aligned}$$

1b $$\begin{aligned} \varepsilon _{exp}^{l,2}&=\sum _{f=f_{min}}^{f_{max}} \left( p^{exp}(f) \right) ^2. \end{aligned}$$

For the following unloading, $\varepsilon _{diff}^{u,2}, \varepsilon _{exp}^{u,2}$, the next part of the data is used and so on. For the overall error, both the differences and the experimental values are summed up and afterwards divided

2 $$\begin{aligned} \varepsilon _p = \sqrt{\frac{\varepsilon _{diff}^{u,1} +\varepsilon _{diff}^{l,2} +\varepsilon _{diff}^{u,2} +\varepsilon _{diff}^{l,3} }{\varepsilon _{exp}^{u,1} +\varepsilon _{exp}^{l,2} +\varepsilon _{exp}^{u,2} +\varepsilon _{exp}^{l,3} }}. \end{aligned}$$

The calculated values of $\varepsilon _p$ are shown in Table 2 together with the bulk stiffness. For the calculation of the bulk stiffness, two lines are fitted at the second and third loading in the stress-strain diagram between 18 kPa and 55 kPa (minimal and maximal applied stresses were 11 kPa and 66 kPa). The average between the slope of these lines is the bulk stiffness, *B*. The calculated error values in Table 2 are all very low, but it can be clearly seen that the simulation result, obtained from the first initial setting, shows a bigger deviation from the measurement data than the others. This effect cannot be seen from the bulk stiffness values. The bulk stiffness calculated from the experimental curve is $B=9.4~\hbox {MPa}$. Despite the good agreement of the simulation results in Fig. 4 and the low calculated errors, the deviation in the bulk stiffness values is considerably larger. Therefore, the bulk stiffness is considered to be a suboptimal measure for comparing experimental and simulation results.

### Direct shear tests

In the simulation of the direct shear test, the normal load is applied on the sample using the same steel loading plate as above by applying a P-control algorithm. After the specified normal load is reached and the packing is at rest, the shearing phase starts by imposing a velocity of 1 mm/s on the lower ring. The shearing force is calculated as the sum of the forces on all walls of the lower ring and the bottom plate in the direction of the shearing.

**Fig. 5:**
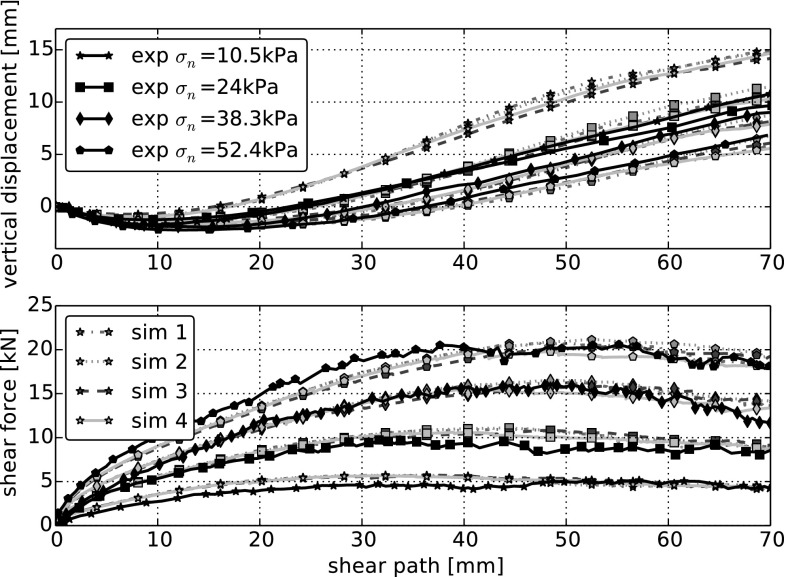
DEM simulations of direct shear tests using the Hertz–Mindlin model for four different initial configurations and four *normal stresses*. All simulations are conducted with $E=50~\hbox {MPa}$ and $\mu =0.5$. Experimental data are taken from [5]

In the direct shear test, the influence of the model parameters *E* and $\mu$ is known from literature, compare e.g. [16]. In a simulation, the Young’s modulus *E* determines the initial stiffness in the shear force–shear path diagram. With increasing *E* also the initial stiffness increases. The friction coefficient $\mu$ influences the maximal or residual shear force obtained in the simulation. The maximal or residual shear force increases with increasing $\mu$. Using this knowledge, a good fit between simulation and measurements is obtained using the parameters $E=50~\hbox {MPa}$ and $\mu =0.5$. These values vary significantly from the values chosen for the compression test ($E=600~\hbox {MPa}$ and $\mu =0.3$). In Fig. 5 simulation results of four different initial configurations are shown for four different normal stresses. The scatter in simulated shear force and vertical displacement is very low (for each normal stress). The simulated shear force approximates the measured force very well. At the lowest applied normal stress, the vertical displacement is too high. For all other normal stresses, simulation and measurement are in much better agreement.

In the direct shear test, two different relative errors will be defined: one for the shear force and one for the vertical displacement. In the equations below, *x* is the shear path, which runs from 0 to 70 mm and $\sigma _n$ is the applied normal stress. Then, $v^{exp}_{\sigma _n}(x)$ and $v^{sim}_{\sigma _n}(x)$ are the vertical displacement at normal stress $\sigma _n$ and shear path *x* for the measurement and the simulation respectively. The shear force $sf^{exp}_{\sigma _n}(x)$, $sf^{sim}_{\sigma _n}(x)$ is denoted analogously. Then, the relative error for the vertical displacement, $\varepsilon _v$ and the shear force $\varepsilon _{sf}$ is formulated and the error is summed up for all four levels of applied normal stress and divided by four.

3a $$\begin{aligned} \varepsilon _v&= \frac{1}{4} \sum _{\sigma _n \in \{ 10.5, 24, 38.3, 52.4\}} \sqrt{ \frac{\sum _{x=0}^{70} \left( v^{exp}_{\sigma _n}(x) - v^{sim}_{\sigma _n}(x)\right) ^2}{\sum _{x=0}^{70} \left( v^{exp}_{\sigma _n}(x) \right) ^2}} \end{aligned}$$

3b $$\begin{aligned} \varepsilon _{sf}&= \frac{1}{4} \sum _{\sigma _n \in \{ 10.5, 24, 38.3, 52.4\}} \sqrt{\frac{\sum _{x=0}^{70} \left( sf^{exp}_{\sigma _n}(x) - sf^{sim}_{\sigma _n}(x)\right) ^2}{\sum _{x=0}^{70} \left( sf^{exp}_{\sigma _n}(x) \right) ^2}} \end{aligned}$$

In Table 3 the calculated errors are given for the simulations with the four different initial configurations together with the bulk friction angle, $\phi$, and the angle of dilation, $\psi$. The error for the vertical displacement is bigger than the shear force error. This is caused by a relatively high error in the vertical displacement for the lowest applied normal stress, which caused for all four initial configurations more than 50% of the total error in vertical displacement. The error for the shear force is lower, as was to expected by the good fit to the experimental data shown in Fig. 5. For the computation of $\phi$, a residual shear force is calculated as median of the shear force between 40 and 60 mm shear path. The slope of a line fitted through the residual shear forces plotted over the four levels of applied normal force allows the calculation of the bulk friction angle. The angle of dilatancy, $\psi$, is calculated as the average of the angles of lines fitted to the vertical displacement between 40 and 60 mm shear path. From the experimental data $\psi ^{exp}=12.6^{\circ }$ and $\phi ^{exp}=54.1^{\circ }$ is calculated. The values of $\psi$ obtained from the simulations are a little bit higher than the experimental ones. This can be seen in the upper part of Fig. 5 for the two lower levels of applied normal stress. The values of bulk friction angle are already quite close to the experimental value. Finally it can be concluded that both the calculated errors as well as the characteristics $\psi$ and $\phi$ confirm the very good fit of the shear force to the experimental data and the relatively good fit of the vertical displacements. It is important to keep in mind that the defined errors include more information, e.g. deviations in the initial stiffness of the shear force curve will be considered.

**Table 3:** Simulations of the direct shear tests using the Hertz–Mindlin model with different initial configurations: relative sum of squares error for vertical displacement, $\varepsilon _v$, and shear force, $\varepsilon _{sf}$, bulk friction angle, $\phi$, and angle of dilation, $\psi$. For comparison, $\phi$ and $\psi$ calculated from the experimental data are also given

Initial conf.	1	2	3	4	Experiment
$\varepsilon _v$ [−]	0.30	0.29	0.24	0.26	−
$\varepsilon _{sf}$ [−]	0.11	0.12	0.12	0.10	−
$\psi [^{\circ }]$	14.3	14.5	14.2	13.5	12.6
$\phi [(^{\circ }]$	53.7	54.6	52.1	51.3	54.1

### Need for contact model modifications

In the preceding subsections, it is shown that the simulation can approximate well the experimental results when only *one type of test* is considered. Keeping in mind how strong the geometry of the crushed rock is simplified, the quality of the approximation is surprising. As it will be detailed below, the conclusion, which is to be drawn here, is that using the simplified Hertz–Mindlin contact model together with the simple particle shape representation, it will not be possible to find parameters *E* and $\mu$ such that *both cyclic compression and monotonic shear test* will be approximated well. In this work, only the simplified particle representation with clumps of two equi-sized spheres is considered. To clarify the influence of a very accurate geometry representation on the observed restrictions of the Hertz–Mindlin model, a detailed investigation would be needed which is outside of the scope of the current study. However, it will be seen later in this section that in [6] similar findings are obtained while using a more realistic but still simplified particle representation via potential particles.

**Fig. 6:**
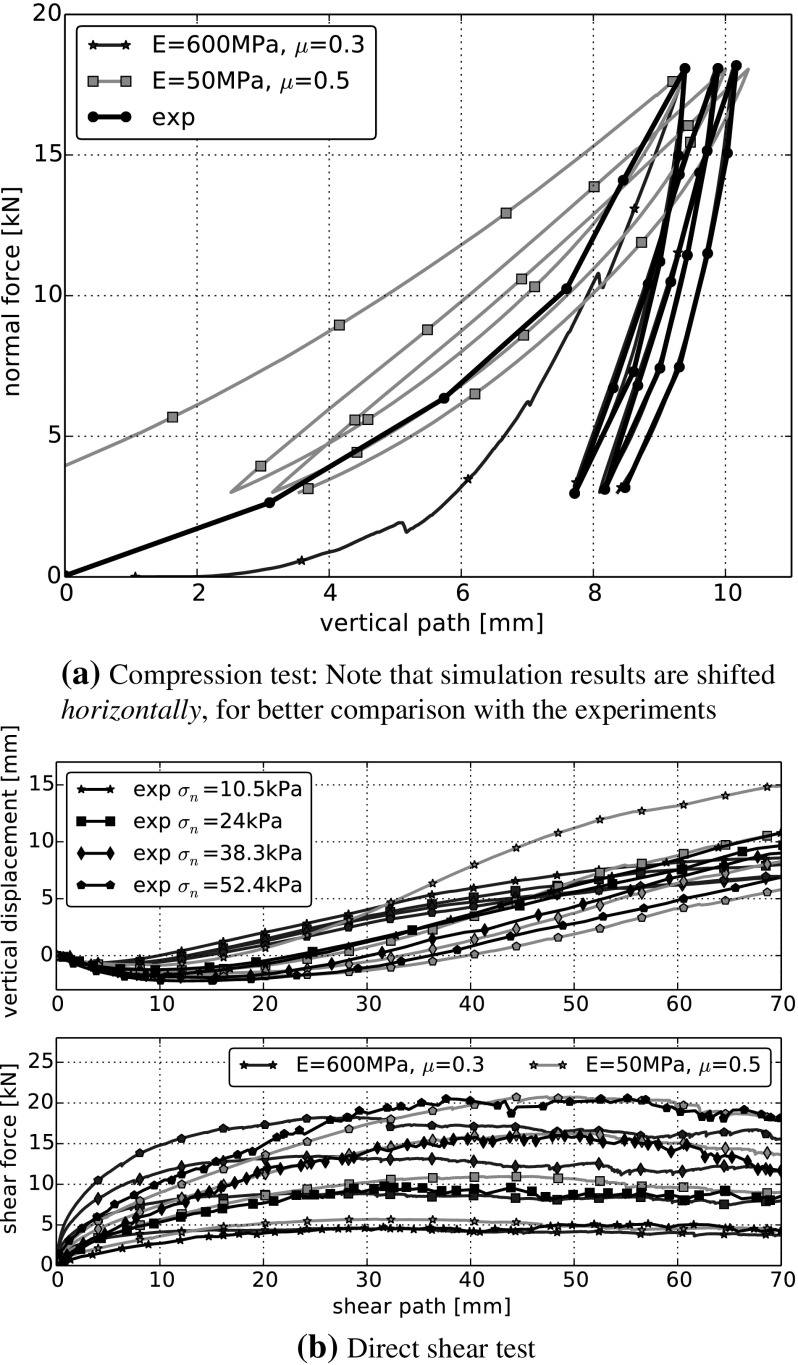
DEM simulations with Hertz–Mindlin model for four different initial configurations. For the simulations parameter sets ($E=600~\hbox {MPa}$, $\mu =0.3$) and ($E=50~\hbox {MPa}$, $\mu =0.5$) were used. Experimental data taken from [5]

In Fig. 6 both the compression and the direct shear test are simulated with the two sets of parameters found: ($E=600~\hbox {MPa}$, $\mu =0.3$) give a good fit for the compression test and ($E=50~\hbox {MPa}$, $\mu =0.5$) for the direct shear test. In Fig. 6a simulations using $E=50~\hbox {MPa}$, $\mu =0.5$ are not at all in agreement with the experimental values, regarding bulk stiffness or the area enclosed by the single loading-unloading cycles. The same holds true in Fig. 6b for the simulations using $E=600~\hbox {MPa}$, $\mu =0.3$. Here the bulk shear stiffness, the residual shear force and the compression-dilation behaviour do not agree with the experiments.

**Table 4:**
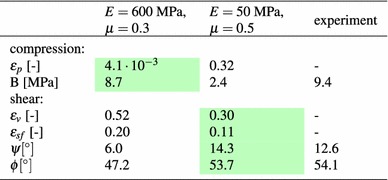
Calculated errors and data characteristics for DEM simulations of compression and direct shear test using the Hertz–Mindlin model with parameter sets ($E=600~\hbox {MPa}$, $\mu =0.3$) and ($E=50~\hbox {MPa}$, $\mu =0.5$). For comparison, characteristics calculated from the experimental data are also given

The calculated errors and data characteristics are given in Table 4, where values indicating a good fit have a coloured background. For the compression test, both the calculated error as well as the bulk stiffness show the poor agreement between the simulation with ($E=50~\hbox {MPa}$, $\mu =0.5$) and the experimental data. In the direct shear test, the calculated errors as well as the angle of dilation indicate clearly that the simulations using ($E=600~\hbox {MPa}$, $\mu =0.3$) are in bad accordance with the experiments. Interestingly, this cannot be seen from the bulk friction angle. These simulations result in a much too high initial stiffness and in considerably too low values of residual shear force, compare Fig. 6. The problems with the initial stiffness cannot be detected using the bulk friction angle. Even the big differences in the residual shear force reduce the bulk friction angle only by roughly 10%. Therefore, the bulk friction angle is considered unsuitable for measuring the quality of fit between simulated and experimental shear force.

From the results presented above, it can be seen that using the simple particle shape representation together with the Hertz–Mindlin contact law, it is not possible to find parameters *E* and $\mu$ such that both the direct shear test and the cyclic compression test are approximated well. For this problem there are two probable causes. First, it is possible that the two-sphere clumps used in the simulations are a too strong simplification of the real particles’ shape or that particle size distribution plays an important role. Second, in the experiments there could occur physical phenomena at contact level, which cannot be represented by Hertz–Mindlin law based on linear material behaviour.

Regarding the first point of particle shape representation and size distribution, it is helpful to consider the work of Harkness et al. [6]. They considered monotonic and cyclic triaxial tests of scaled railway ballast. Here, scaled ballast means a reduction in size and a comparison with ballast of original size was done in previous works, see [13, 19, 20]. In their DEM simulations the simplified Hertz–Mindlin contact law is used together with complex shaped potential particles which matched the particle size distribution of the tested railway ballast. In [13], close agreement between purely monotonic triaxial tests and simulations using a similar setup could be achieved. In [6], monotonic and cyclic triaxial tests are considered at different confining pressures. It was shown that the Young’s modulus (or shear modulus) used for the simulations of monotonic triaxial tests, is considerably too low to reproduce the residual modulus seen in the cyclic triaxial tests. Thus, Harkness et al. faced a similar problem, as occurred in this work, when both monotonic (direct shear) and cyclic (compression) tests are to be simulated. Because particle shape and size distribution are already taken into account in a complex representation in [6], further investigations of this topic are not considered useful in this work.

On the contrary, it is assumed here, as it is in [6], that physical phenomena not included in the Hertz–Mindlin model cause the deviations between simulation and experimental results. Particle breakage does not seem to play an important role: in [6], confining pressures up to 200 kPa were applied and the particle size distribution remained nearly unchanged. Particle damage, e.g. breakage of edges, can be expected to occur and can explain the differences in material behaviour in monotonic and cyclic tests. In monotonic tests and in the first cycle of the cyclic tests, damage increases continuously, leading to an apparent soft (or apparent plastic) material response. In cyclic tests, the following cycles show a much stiffer response, because particle damage already occurred at the first cycle. During these follow up cycles, damage still occurs, due to particle rearrangements, but its extent can be expected to be much smaller than in the first cycle.

In [6], similar thoughts motivated the modification of the used Hertz–Mindlin contact law, to take into account this particle damage (in form of ideal plastic behaviour). This contact law is discussed in the next section in detail. It will be shown in this work that using this modified contact law experimental results from both the compression test and the direct shear tests can be predicted well using only one set of parameters. Moreover, the differences in particle damage in the monotonic direct shear test and the cyclic compression test will be analysed, which will further justify the above argumentation.

## Conical damage model

### Normal direction

In [6], Harkness et al. presented the Conical Damage Model (CDM) for the normal contact, which will be used also in this work. The elastic part of the material behaviour is modelled via the Hertz law. Additionally, a kind of ideal plasticity is is introduced to model damage at a contact (e.g. to take into account edge breakage). In [6], the contact between a sphere and a plane is imagined as the contact between an asperity on the sphere and the plane. The asperity is conical in shape and is described by an initial radius and an opening angle, specified by material parameters. While the contact is elastic in the first place, it becomes plastic when a certain stress is reached. Then, damage occurs and the radius of the asperity grows, leading to an increased area of contact and thus to a lower stress. In the parametrised model in [6], the initial radius of the asperities is 12 mm, which is clearly bigger than any real asperity.

In this work, a slightly different formulation of the CDM is used and an efficient algorithm for an accurate and non-iterative solution will be given. In this interpretation, there exists an elastic regime, where each contact behaves as in the classical Hertz–Mindlin model. When a certain stress is reached, the surface of the DEM particle is imagined to flatten locally as damage occurs. As described above, this flattening corresponds to a larger contact area and thus to a lower stress. By this interpretation the number of needed parameters is reduced by one (initial radius of the asperity) and the behaviour of the new contact model in the elastic regime is identical to the classical Hertz–Mindlin model. A detailed description is given below.

The model is formulated here for sphere-sphere contact, but plane-sphere contact can be treated analogously. The geometric overlap at the contact, which is calculated by the DEM software, $\delta _{\textsc {DEM}}$ is split in an elastic part, $\delta _{el}$, and a plastic part, $\delta _{pl}$:

4 $$\begin{aligned} \delta _{\textsc {DEM}}= \delta _{el} + \delta _{pl}, \end{aligned}$$

where $\delta _{pl}$ is initialised with zero when the contact is created. In the purely elastic regime, Hertz’ contact law is used for the calculation of the normal force:

5 $$\begin{aligned} F_n = \frac{4}{3} E^* \sqrt{R} \left( \delta _{el}\right) ^\frac{3}{2}, \end{aligned}$$

where $E^*$ is the equivalent Young’s modulus and *R* is the current radius in the contact which is initialized with the equivalent radius $R^*$. According to Hertz’ law, the maximal stress at the contact, $\sigma _0$, can be calculated as

6 $$\begin{aligned} \sigma _0 =\frac{2 E^*}{ \pi } \sqrt{ \frac{\delta _{el}}{R}}. \end{aligned}$$

**Fig. 7:**
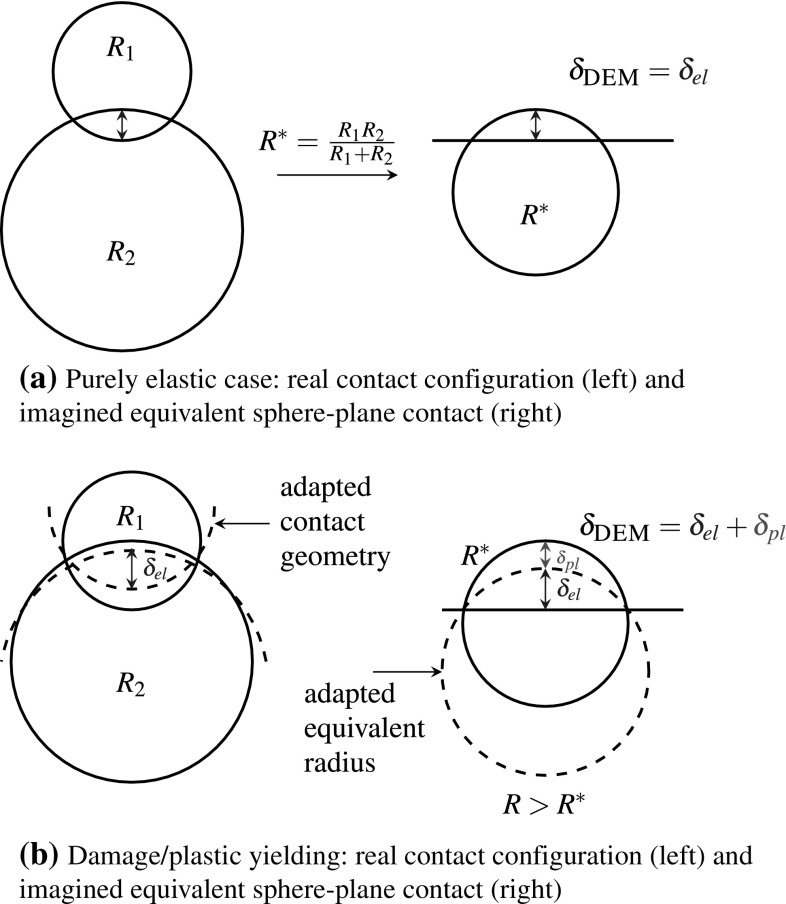
Conical damage model for a sphere–sphere contact

In the purely elastic regime, this stress is smaller than or equal to a pseudo maximal compressive strength, $\sigma _{{\mathrm{max}}}$. This parameter is not the real compressive strength of the material, deviations are caused by simplifications in geometry representation and surface properties. In the other loading case, i.e. $\sigma _0 > \sigma _{{\mathrm{max}}}$, the stress is too high for the material to be carried and damage/plastic yielding occurs. In this model, the sphere in contact will flatten, thus *R* increases as well as $\delta _{pl}$. In this way, the maximal stress at the contact will be reduced to $\sigma _{{\mathrm{max}}}$. A schematic drawing of this principle can be found in Fig. 7 for the contact between two spheres. Figure 7a shows the case of the initial purely elastic deformation. The sphere-sphere contact is transferred to an equivalent sphere-plane contact. Before plastic yielding, the radius of the imagined sphere is the equivalent radius, $R^*$. The case when the maximal stress in the contact exceeds $\sigma _{{\mathrm{max}}}$ is shown in Fig. 7b. Now, the radius of the imagined sphere grows, as it is drawn in Fig. 7b. The enlarged radius of the imagined sphere can be understood as the equivalent radius of two larger spheres, which are in contact. Locally, the two spheres in contact are thought have flattened, which corresponds to a larger contact area and to an admissible stress $\sigma _0 = \sigma _{{\mathrm{max}}}$. How the total overlap $\delta _{\textsc {DEM}}$ is split into the elastic and the plastic part is also shown on the right side of Fig. 7b. Finally, the relation between *R* and $\delta _{pl}$ has to be given. In [6] it is derived from a geometric relation involving the opening angle of the conical asperity, $\alpha$. This assumption leads to a linear relation between both quantities:

7 $$\begin{aligned} \delta _{pl} =(R - R^*) \beta , \end{aligned}$$

where the material parameter $\beta$ relates to $\alpha$ as: $\beta = \frac{1-\sin (\alpha )}{ \sin (\alpha )}$. The new value of *R* can be calculated using the yield condition

8 $$\begin{aligned} \sigma _{{\mathrm{max}}}=\sigma _0 =\frac{2 E^*}{ \pi } \sqrt{ \frac{\delta _{el}}{R} }. \end{aligned}$$

When Eqs. (4) and  (7) are inserted above, the obtained expression can be solved for *R*:

9 $$\begin{aligned} R=\frac{ \delta _{\textsc {DEM}}+ R^* \beta }{ \left( \frac{ \sigma _{{\mathrm{max}}}\pi }{2 E^*}\right) ^2 + \beta }. \end{aligned}$$

With this equation it is possible to solve the model accurately without the need of an iterative procedure. Keeping in mind the high number of contacts in a DEM simulation, this is a clear advantage of the CDM model, as many models for plasticity at contacts need to be solved iteratively, compare e.g. [21]. The algorithm for the calculation of the model is given below, see Algorithm 1. As mentioned before, this model accounts for additional physical phenomena (yielding) and allows therefore to improve the quality of DEM models.
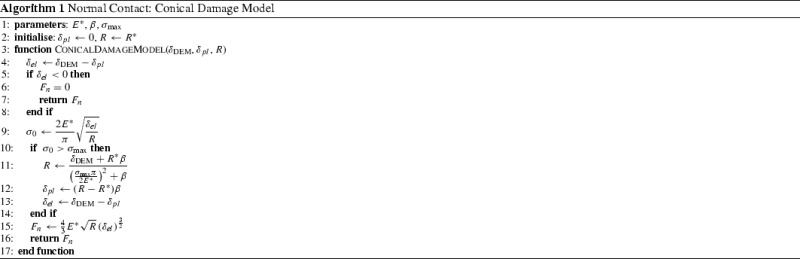


### Tangential direction

In tangential direction the simplified no-slip Mindlin law will be used. For consistency, here also the increased radius of the Hertzian contact described above will be used. In time step *k* the trial or pre-sliding shear force is denoted by $F^k_{t,t}$ and is calculated incrementally using the last time step’s value:

10 $$\begin{aligned} F^k_{t,t} = F^{k-1}_{t} + \varDelta F_{t,t} \qquad \varDelta F_{t,t}= 8 G^* \sqrt{R \delta _{el}}\, \varDelta \delta _t, \end{aligned}$$

where $\varDelta \delta _t$ is the increment of the tangential displacement and $G^*=\left( \frac{2-\nu _1}{G_1} + \frac{2-\nu _2}{G_2}\right) ^{-1}$ is the equivalent shear modulus calculated from the contact partners shear modulus and Poissons ratio. For simplicity, the index of the time step will be skipped from now on. When Coulomb’s law with a constant coefficient of friction, $\mu$, is used, the tangential force is obtained as follows:

11 $$\begin{aligned} F_{t}=\left\{ \begin{array}{ll} F_{t,t}&{} \text{ if } F_{t,t}\le \mu F_n\\ \mu F_n&{} \text{ otherwise } \end{array} \right. \end{aligned}$$

To the authors’ best knowledge, a dependency of the coefficient of friction on the applied normal pressure in DEM simulations was first introduced in their own works [17, 22] and [23]. In tribology, it is known that the friction coefficient in a contact is not constant, as it is assumed in Coulomb’s law, but depends on several factors, such as contact normal load, relative motion, surface roughness, contact temperature and contact conditions (dry, wet, lubricated, ...), etc. In the literature there exist works, where in direct shear tests a dependency of the bulk friction coefficient on the applied normal pressure can be seen, see e.g [16, 24, 25]. Whether this effect can be seen in experiments will depend on the material, particle shape and applied normal pressure. In [17] and [23], the authors assumed that the observed normal stress dependency of the bulk friction coefficient is caused by tribological effects. Thus, a more tribological tangential contact law is implemented in DEM, where the interparticle friction depends on the current normal stress in the contact. For each contact a (spatially) averaged stress is calculated, by dividing the contact force with the area of the contact:

12 $$\begin{aligned} \sigma _m= \frac{F_n}{R \delta _{el} \pi }. \end{aligned}$$

This stress is used for the calculation of the pressure dependent friction coefficient for this contact

13 $$\begin{aligned} \mu (\sigma _m)= \mu _0 + \frac{c_1}{1+c_2 \sigma _m}, \end{aligned}$$

where $\mu _0 [-], c_1 [-], c_2 [\text{ Pa }^{-1}]$ are model parameters. This formula was first introduced in [26] for wheel–rail (steel–steel) contact. The pressure dependent friction coefficient can then be used in Coulomb’s law, (11). In this adapted formulation of the model the change of the contact area, representing particle damage, is already integrated. The averaged stress in this model cannot be bigger then $\sigma _m\le \frac{2}{3} \sigma _{{\mathrm{max}}}$, which can be seen from combining Eqs. (5) and (8). Thus, in this model the friction coefficient is bounded: $\mu _0 + c_1 \le \mu (\sigma _m) \le \mu _0 + \frac{c_1}{1+c_2 \frac{2}{3} \sigma _{{\mathrm{max}}}}$.

A little later and in an independent approach, in [6] Harkness et al. introduced a friction coefficient, which depends on the normal force on the contact. A normalisation of the normal force via the maximal possible force is formulated, also:

14 $$\begin{aligned} \mu (F_n)= \gamma _0 \left( \frac{F_n}{F_c}\right) ^{\gamma _1}, \end{aligned}$$

where $\gamma _0, \gamma _1$ are dimensionless model parameters and $F_c$ is the critical force, $F_c=\frac{2}{3} \sigma _{{\mathrm{max}}}\pi R \delta _{el}$. Some basic manipulations of the expression $F_n/F_c$ show that the model in Eq. (14) can also be written as:

15 $$\begin{aligned} \mu (F_n)= \gamma _0 \left( \frac{F_n}{F_c}\right) ^{\gamma _1} = \frac{\gamma _0}{\left( \frac{2}{3} \sigma _{{\mathrm{max}}}\right) ^{\gamma _1}} \left( \sigma _m\right) ^{\gamma _1}, \end{aligned}$$

and is very similar to the model in Eq. (13), with the difference that $\mu$ approaches infinity when $F_n$ approaches 0 (and $\gamma _1$ is smaller than zero, which needs to be the case for a declining behaviour).

## Parameter study

The Conical damage model described in the previous section introduces new parameters, whose influence on the simulated bulk behaviour is not clear a priori. When used in a DEM simulation, there are several parameters, which need to be chosen: *E*, $$ \nu $$, $$ \sigma _{{\mathrm{max}}}$$, $$ \beta $$, $$ \mu _0$$, $$ c_1$$, $$ c_2$$. In this section, a parameter study is conducted to better understand the role of the single parameters. A simple but efficient strategy is chosen: based on one set of parameters, the centre point, one parameter at a time is varied. This approach clearly cannot capture any interaction effects between the parameters and it reflects only the behaviour around the centre point. The advantage of this approach is the very limited number of needed simulation runs. An alternative would be to use Design of Experiments together with a fractional design. As the results of the one at a time variation are easier to interpret, this approach is taken here.

In a first step, the friction coefficient, $\mu$, is considered to be constant to reduce the number of investigated parameters. Additional simulations, which are not presented here, showed that $\nu$ had only a very small influence. Therefore, $\nu =0.2$ is used in all simulations from now on. Thus, the parameter study will then be conducted on $E, \sigma _{{\mathrm{max}}}, \beta , \mu$ and the centre point was chosen as: $E=100~\hbox {MPa}$, $\sigma _{{\mathrm{max}}}=3~\hbox {MPa}$, $\beta =0.015$, $\mu =0.4$. In the next step, simulations of compression tests and direct shear tests are conducted, where the parameters are varied separately.

**Fig. 8:**
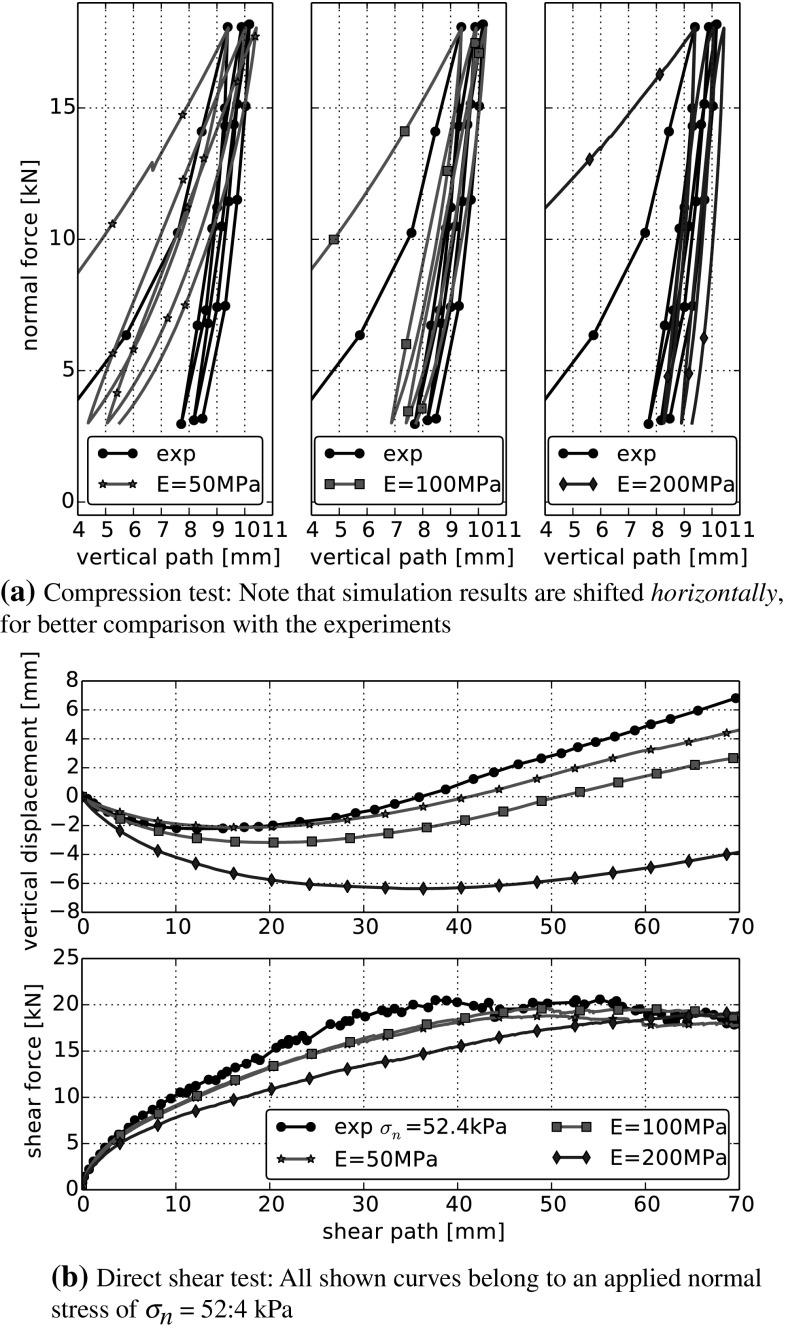
Parameter variation in DEM simulations: Young’s modulus *E*. All other parameters are set to their values of the centre run: $\sigma _{{\mathrm{max}}}=3~\hbox {MPa}$, $\beta =0.015$, $\mu =0.4$. Experimental data taken from [5]

The effect of a variation of the Young’s modulus can be seen in Fig. 8 for the compression and the direct shear test respectively. For the compression test, an increase of *E* results in a considerable increase in the bulk stiffness and reduces the area enclosed by the single loading-unloading loops. In the direct shear test, an increase of *E* results in a lower slope in the shear force—shear path curve (after ca. 5 mm of shear path). Also, more compression takes place together with a decrease in the angle of dilation. When *E* increases, contacts will start to yield earlier. Also *R* increases which results in an increased $\delta _{pl}$ and in a decreased $\delta _{el}$. The mean contact normal force is smaller and due to Coulomb’s law also the tangential force is lower. Therefore, a longer shear path is needed until the same shear force is reached as in simulations with lower Young’s modulus.

**Fig. 9:**
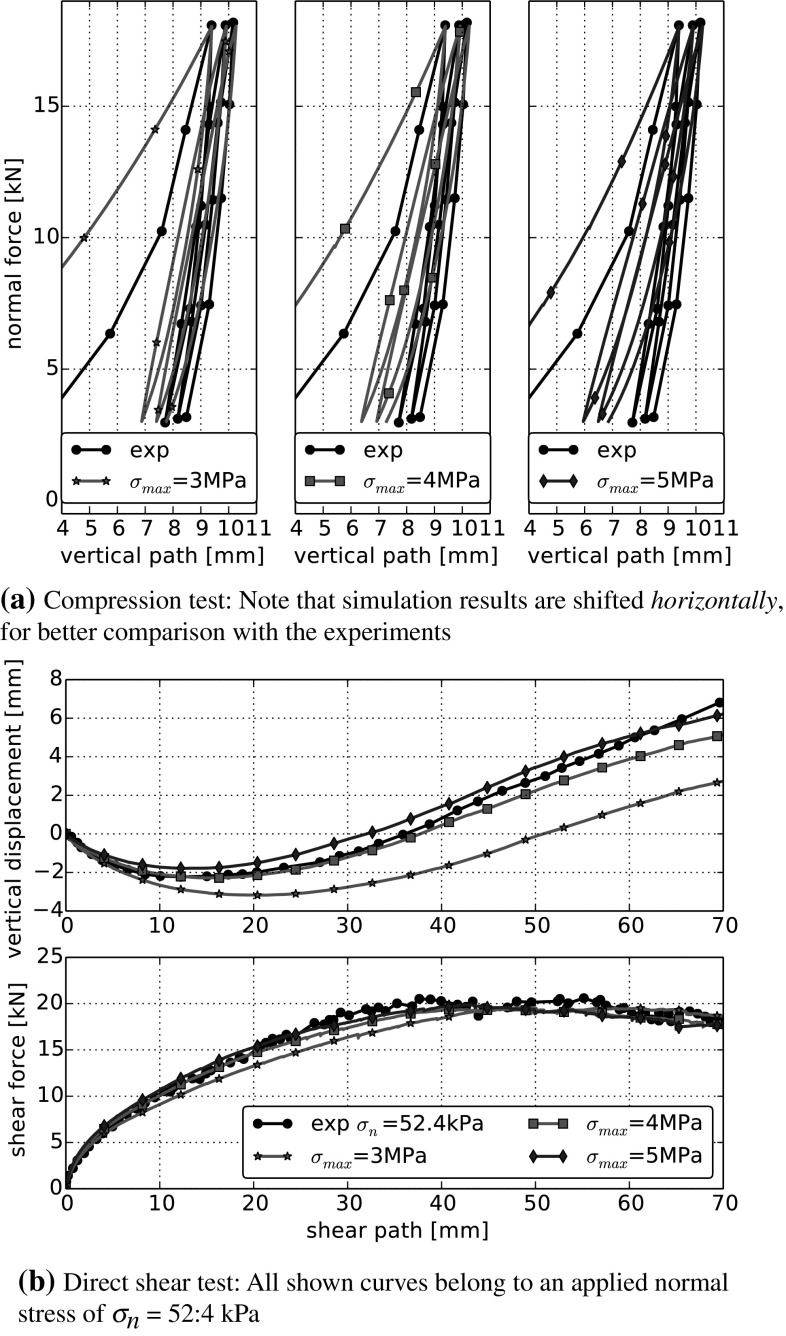
Parameter variation in DEM simulations: maximal compressive strength $\sigma _{{\mathrm{max}}}$. All other parameters are set to their values ofthe centre run: $E=100~\hbox {MPa}$, $\beta =0.015$, $\mu =0.4$. Experimental data taken from [5]

In Fig. 9, the effects of a variation of the maximal compression strength, $\sigma _{{\mathrm{max}}}$, can be seen. An increase of $\sigma _{{\mathrm{max}}}$ slightly reduces the bulk stiffness and increases the area enclosed by the single loading-unloading loops in the compression tests. In the direct shear test, an increase of $\sigma _{{\mathrm{max}}}$ results in a higher slope in the shear force–shear path curve and in less compression, while the angle of dilation remains nearly constant. With a higher compressive strength, less contacts will show plastic yielding or damage. If the compressive strength was chosen as ‘infinity’, then the model would be identical to the purely elastic Hertz–Mindlin contact law.

**Fig. 10:**
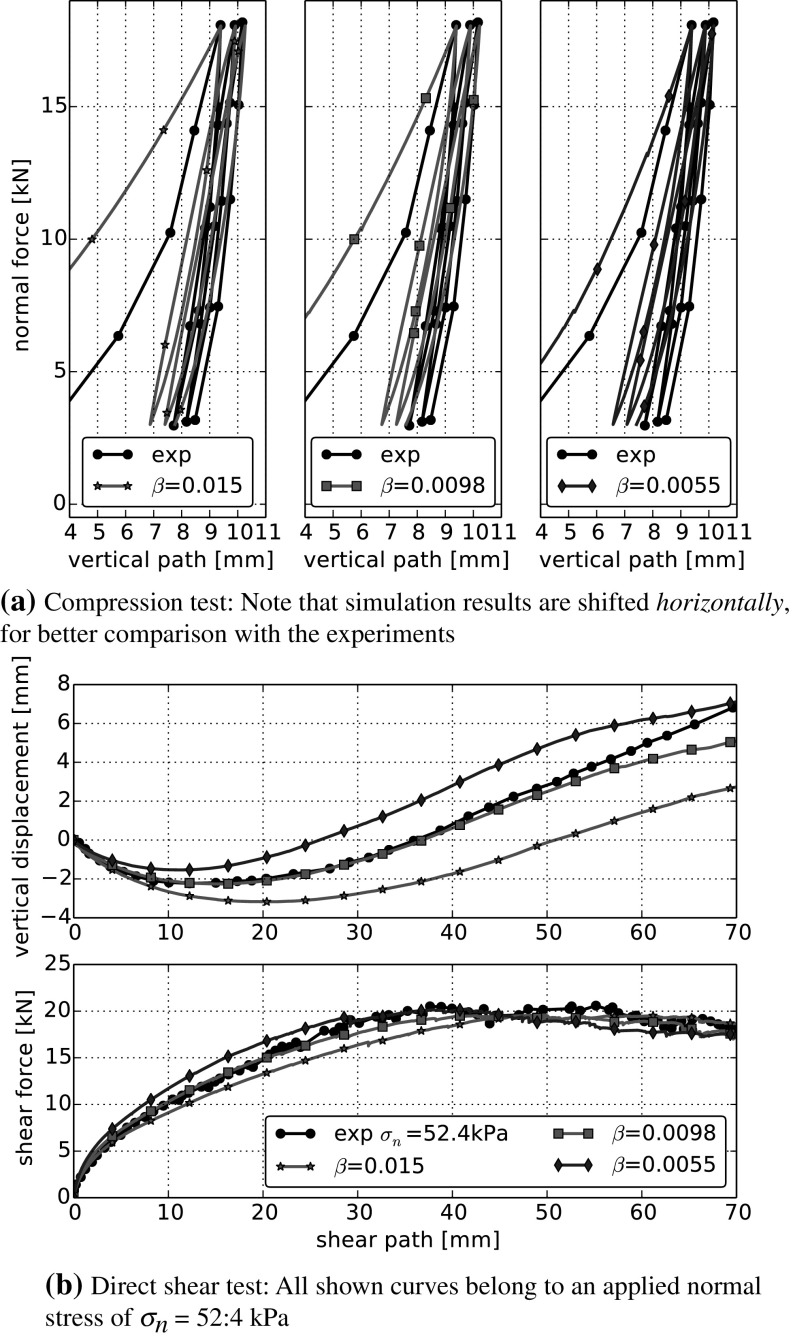
Parameter variation in DEM simulations: $\beta$. All other parameters are set to their values of the centre run: $E=100~\hbox {MPa}$,$\sigma _{{\mathrm{max}}}=3~\hbox {MPa}$, $\mu =0.4$. Experimental data taken from [5]

The variation of $\beta$ is shown in Fig. 10. Around the chosen centre point, an increase of $\beta$ has the same effect on the bulk behaviour as an increase of $\sigma _{{\mathrm{max}}}$. While $\sigma _{{\mathrm{max}}}$ determines, when a contact starts yielding, $\beta$ controls the increase of *R* during yielding. The surprising similarity can not be deduced from the model equations and may not be present for points in parameter space far away from our centre point.

**Fig. 11:**
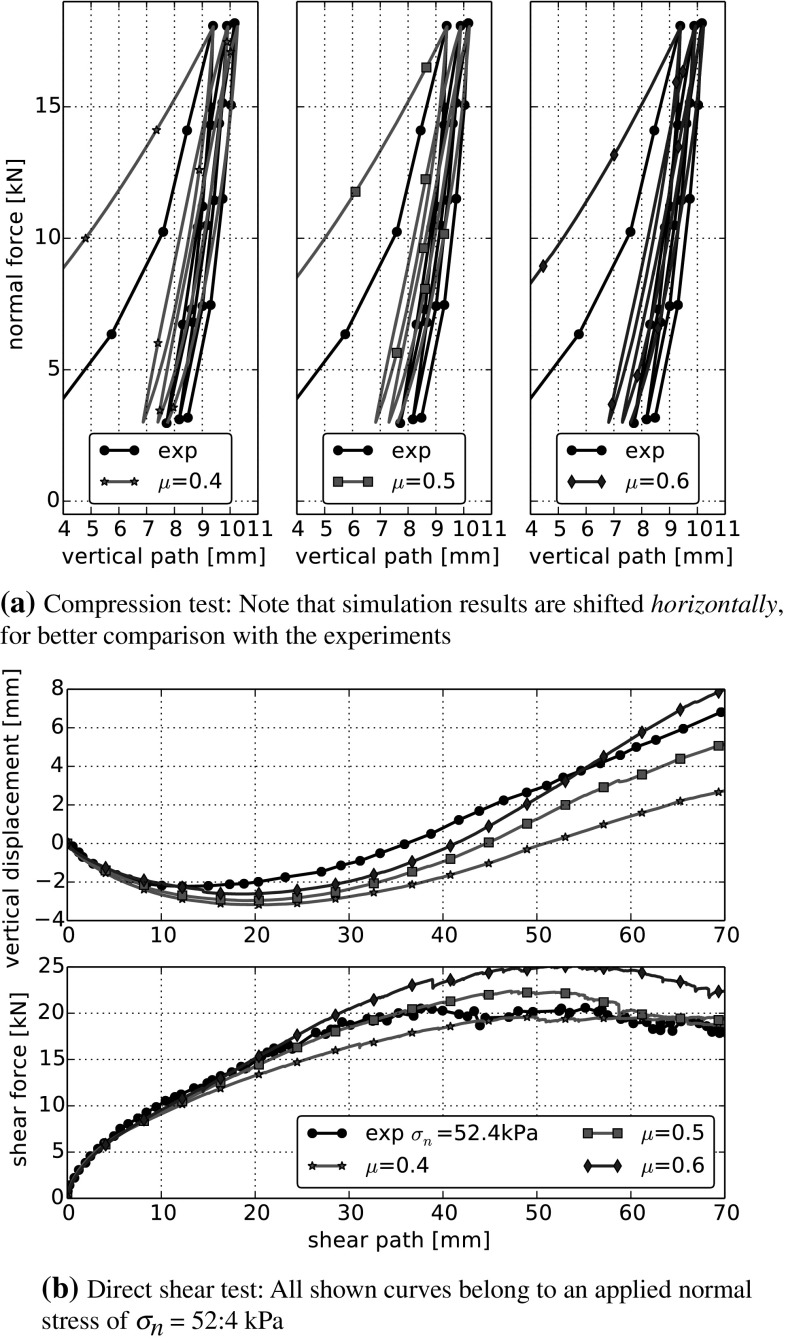
Parameter variation in DEM simulations: constant friction coefficient $\mu$. All other parameters are set to their values of the centre run: $E=100~\hbox {MPa}$, $\sigma _{{\mathrm{max}}}=3~\hbox {MPa}$, $\beta =0.015$. Experimental data taken from [5]

Finally, the variation of the coefficient of friction is shown in Fig. 11. This is the only considered parameter, which is not part of the normal contact model. In the compression test, an increase of $\mu$ reduces the area enclosed by the single loading-unloading loops, while the bulk friction remains nearly constant. In the direct shear test, an increase of $\mu$ becomes noticeable only after 20 mm of shear path. After this, it increases the shear force and the angle of dilation. This behaviour is equal to the one seen in simulations using the classical Hertz–Mindlin contact law.

## Comparison with experiments

**Fig. 12:**
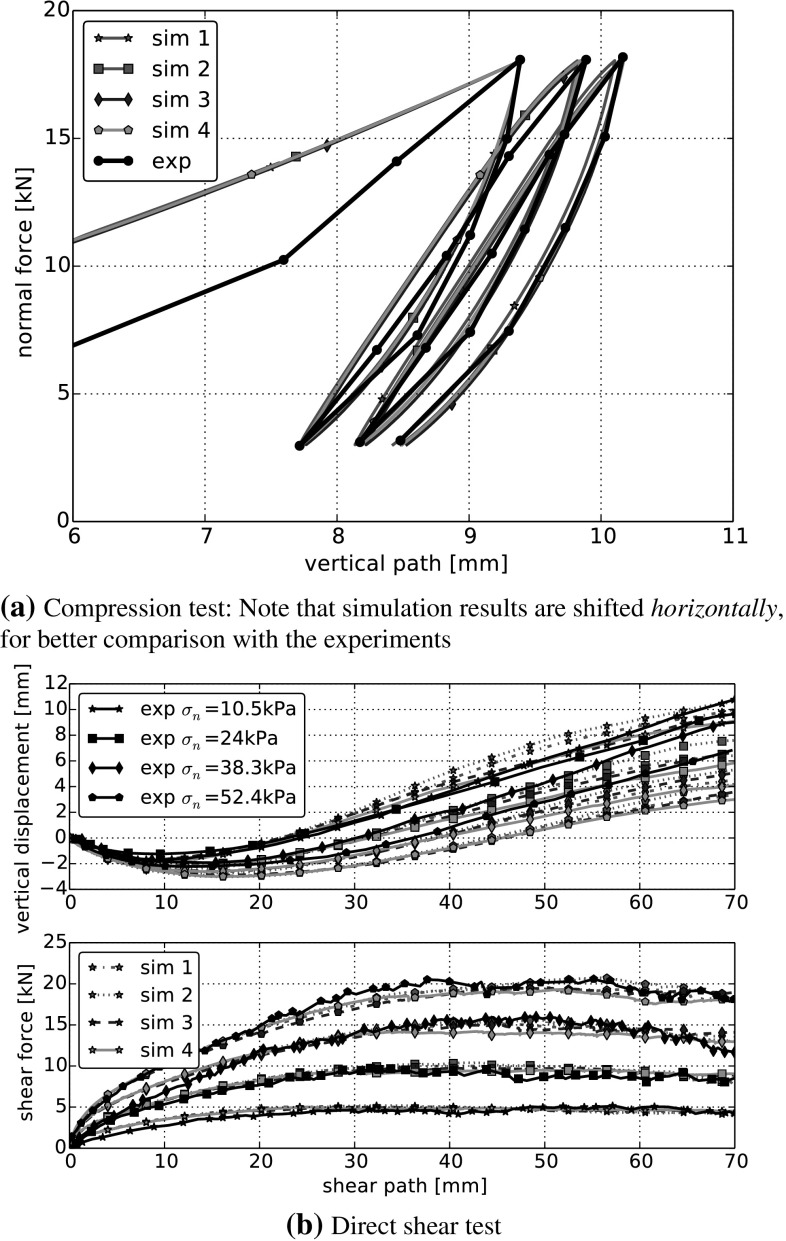
DEM simulations with CDM model for four different initial configurations. Used parameters: *E*=200 MPa, $\sigma _{{\mathrm{max}}}=5~\hbox {MPa}$, $\beta =0.015$, $\mu =0.4$. Experimental data taken from [5]

The knowledge gained on the effects of the single model parameters can now be used to fit the simulation results to the experimental ones. As the effects of $\sigma _{{\mathrm{max}}}$ and $\beta$ are similar around the centre point, it should be noted that it might not be possible to obtain unique values for these two parameters. In general, an optimisation software could be used for parameter identification. As the centre point of the previous parameter study gives already relatively good results, a few ‘try and error’ iterations were enough to obtain a good fit between experiments and simulations. A comparison can be seen in Fig. 12 for four different initial configurations. For all simulations the following set of parameters was used: $E=200~\hbox {MPa}$, $\sigma _{{\mathrm{max}}}=5~\hbox {MPa}$, $\beta =0.015$, $\mu =0.4$. The calculated errors together with B, $\psi$ and $\phi$ are given in Table 5. In the simulations of the compression test, the bulk stiffness, the form of the hysteresis and the path increment of the single cycles agree very well. Therefore, also the calculated error is very low. The calculated bulk stiffness nearly coincides with its experimental value of 9.4 MPa. In the simulations of the direct shear test, the shear force–shear path curve is approximated well for all levels of applied normal stress. The calculated error is very low, while the bulk friction values are slightly lower than the experimental value of $\phi ^{exp}=54.1^{\circ }$. The compression/dilation behaviour is approximated not quite so good, but the results are still considered acceptable. In the simulations, for higher applied normal stresses there occurs more compression than seen in the experiments. Also the angle of dilation is lower than its experimental value $\psi ^{exp}=12.6^{\circ }$.

**Fig. 13:**
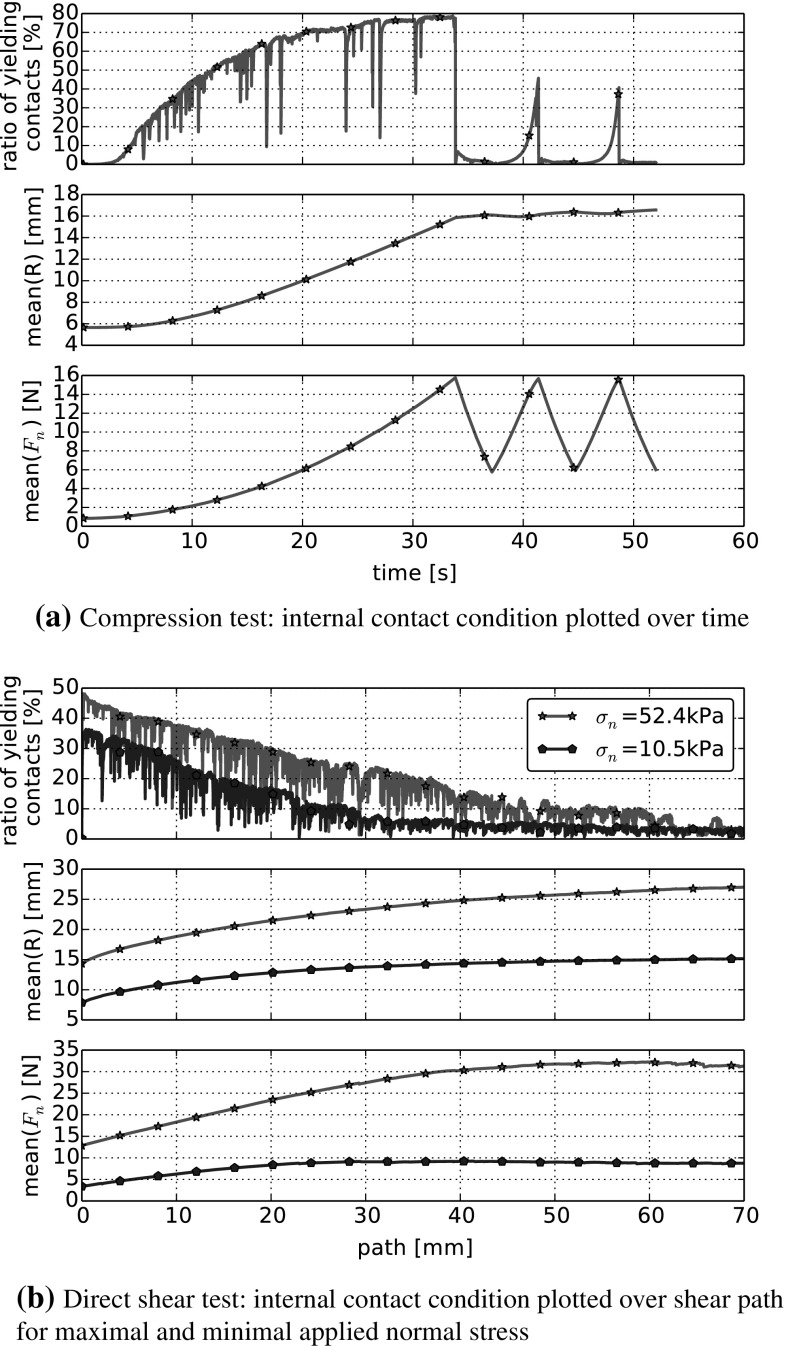
DEM simulations of cyclic compression and direct shear tests with CDM model. Plot of mean internal contact conditions. Used parameters: $E=200~\hbox {MPa}$, $\sigma _{{\mathrm{max}}}=5~\hbox {MPa}$, $\beta =0.015$, $\mu =0.4$

To investigate how the CDM model influences the individual contacts, Fig. 13 shows some internal variables of the direct shear test simulations. In the upper plot, Fig. 13a, the percentage of yielding contacts, the mean value of all contact radii *R* and contact normal forces $F_n$ are plotted over time for the cyclic compression test. During the first loading phase, the ratio of yielding contacts as well as *R* show a strong increase. As the sample is compacted for the first time, the ratio of yielding contacts shows sharp short time reductions, which can be related to particle rearrangement. Both the ratio of yielding contacts and the mean value of all contact radii, *R*, indicate that the most of yielding/breakage of edges occurs during this first loading. In the following unloading, the ratio of yielding contacts reduces to nearly zero and *R* remains constant. During the subsequent re-loading phase, the ratio of yielding contacts remains close to zero and shows a steep increase only shortly before the maximal applied stress is reached, probably due to particle rearrangement and newly created contacts. In the same time, the mean of all contact radii remains nearly constant. The shown behaviour supports the assumption that monotonic and cyclic loading (i.e. first loading cycle and follow up loading cycles), lead to different simulated material behaviour.

In Fig. 13b, the same internal variables are plotted for the monotonic direct shear test over the shear path for the lowest and the highest applied normal stress, i.e. $\sigma _n=10.5$ kPa and $\sigma _n=52.4$ kPa. Before the samples are sheared, the higher normal stress leads to more yielding contacts, bigger contact radii and contact normal forces than the lower applied stress. In this phase, strong force chains are oriented vertically and are distributed over the whole sample. When shearing starts, the strong force chains reorient in a band between the upper and lower ring, which becomes more narrow with growing shear path. Therefore, a reduction of the percentage of yielding contacts can be seen in the upper part of the plot during the shear phase. Both the mean of the contact radii and the normal forces show a qualitative similar behaviour in the middle and lower part of the plot. Their growth is faster in the beginning and reduces with the shear path. Analogue to the shear force curve, plotted in Fig. 12b, both means of radii and normal force reach a steady state earlier for the low applied normal stress than for the high applied normal stress.

**Table 5:** Calculated errors and data characteristics for DEM simulations of compression and direct shear test using the CDM model with parameters $E=200~\hbox {MPa}$, $\sigma _{{\mathrm{max}}}=5~\hbox {MPa}$, $\beta =0.015$, $\mu =0.4$. For comparison, characteristics calculated from the experimental data are also given

Initial conf.	1	2	3	4	Exp.
Compression					
$\varepsilon _p$ [−]	$9.3\cdot 10^{-3}$	$7.8\cdot 10^{-3}$	$7.3\cdot 10^{-3}$	$7.9\cdot 10^{-3}$	−
B [MPa]	9.4	9.3	9.4	9.4	9.4
Shear:					
$\varepsilon _v$ [−]	0.39	0.35	0.39	0.44	−
$\varepsilon _{sf}$ [−]	0.085	0.083	0.082	0.082	−
$\psi [^{\circ }]$	9.9	11.1	9.8	9.5	12.6
$\phi [^{\circ }]$	52.0	53.6	51.9	50.8	54.1

When one keeps in mind how simple the geometry of the crushed rock particles is represented in this work, it is surprising how well simulations and experiments agree. It can be expected that this simplification caused the found material parameters, such as Young’s modulus and compressive strength, to be not close to the literature values specified for rock.

In the results of the direct shear test, there is no dependency of the bulk friction angle of the experiments on the applied normal stress. This is different to the triaxial tests presented in [6]. Due to this reason, the simulations are conducted with constant coefficient of friction.

In contrast to the simulation with the classical Hertz–Mindlin contact law, using the CDM model in normal contact, makes it possible to approximate the experiments of compression and direct shear test with the same set of parameters.

## Conclusions

In this work, DEM simulations of cyclic compression tests and directs shear tests of crushed rock are conducted. Experimental data for comparison is available from literature, see [5]. For computational efficiency, in the simulations the geometry of the crushed rock particles is represented in a very simple approach by two clumped spheres. Works from the literature, compare [4] or [5], show that it is possible to obtain (qualitatively) similar results to experimental measurements using very simple geometry representations. DEM simulations using the simplified Hertz–Mindlin contact law are conducted for the cyclic compression tests and the direct shear tests. For the modelling of railway ballast, it is important to compare simulation and experimental results using the entire curves and not to reduce them to characteristics, such as bulk stiffness, angle of dilation and bulk friction angle. In the compression test, the information of settlement between cycles and the shape of the single loading loops is lost, when only the bulk stiffness is considered. In the direct shear test, the initial stiffness of the shear force–shear path curve is important for track stiffness. This information is not included in the bulk friction angle. Therefore, in this work the entire curves are compared visually and quantitative error measures are introduced, which are based on the sum of squares. Although the Hertz–Mindlin contact model is used successfully to model granular material throughout literature, there exist also works, where the contact model needs to be adapted, compare Sect. 2. It is shown in this work that using the simplified particle representation, the Hertz–Mindlin contact model can be parametrised such that simulation results agree with the experimental measurements for either *one* of the two tests but *not both* at the same time. This finding is in agreement with the work of Harkness et al. [6], which face the same problem when using more realistic shaped potential particles to simulate monotonic and cyclic triaxial tests of railway ballast. Despite this agreement, the stated limitation of the Hertz–Mindlin model might depend on the accuracy of geometry representation.

A modification of the used contact model, accounting for additional physical phenomena, is one possibility to solve this problem. For the normal contact, the conical damage model (CDM), presented in [6], is new interpreted and an efficient algorithm for its accurate solution is presented. For the tangential contact, works of the authors themselves [17, 23] and of Harkness et al. [6] are very similar, when a dependency of the interparticle friction coefficient on the local contact force/stress is introduced.

In a parameter study for the Comical Damage Model, the effects of the single model parameters are shown. With this knowledge a parametrisation of the model is possible. The simulations conducted with the parametrised CDM model show that good accordance with *both* cyclic compression tests and monotonic direct shear tests can be obtained using only *one set of parameters*. The quality of the approximation is surprisingly high considering the simple geometry representation of the crushed rock chosen in this work.
